# Psychological Symptoms in Children with Radioulnar Synostosis: Associations with Self-Appraised Resilience, Parental Anxiety and Measured Elbow and Shoulder Function in a Cross-Sectional Multi-Informant Study

**DOI:** 10.3390/children13101371

**Published:** 2026-10-09

**Authors:** Zeynep Seda Albayrak, Gorkem Kayis, Melisa Ercan, Sefa Seluk, Kutalmis Albayrak

**Affiliations:** 1 Department of Child and Adolescent Psychiatry, School of Medicine, Koç University, Istanbul 34010, Türkiye; 2 Koç University Research Center for Translational Medicine (KUTTAM), Istanbul 34010, Türkiye; 3 Department of Orthopaedics and Traumatology, Baltalimanı Bone Diseases Training and Research Hospital, Istanbul 34470, Türkiye; drgorkemkayis@gmail.com (G.K.); drmelisademir@gmail.com (M.E.); drsefaseluk@gmail.com (S.S.); kutalmisalbayrak@gmail.com (K.A.)

**Keywords:** radioulnar synostosis, paediatric orthopaedics, parental distress, anxiety, resilience, multi-informant assessment, quality of life, congenital upper-limb difference

## Abstract

Background/Objectives: Radioulnar synostosis restricts forearm rotation and is often said to carry psychosocial consequences. That it does so is widely assumed; what has not been tested is whether the extent of those consequences tracks the measures of limb function recorded at follow-up—the assumption implicit in monitoring these children with functional scores alone. We measured psychological symptom scores in affected children and examined what they were related to: the orthopaedic measures available, the aetiology of the synostosis, the child’s own resilience, or the mental state of the parent. Methods: Twenty-four children aged 9–17 years with unilateral, non-syndromic radioulnar synostosis (10 post-traumatic, 14 congenital) attended a single visit at which an orthopaedic examination and psychometric assessment were carried out together. Children completed standardised measures of anxiety and depression, quality of life and self-appraised resilience. Parents provided proxy reports of the child’s anxiety and depressive symptoms and quality of life and, separately, reported their own symptoms on the DASS-21. All analyses were exploratory. Results: Quality of life fell below the international reference mean (KIDSCREEN-10 T-score 43.1, 95% CI: 40.5 to 45.6). Symptom scores were associated with the child’s self-appraised resilience (rho = −0.84, 95% CI: −0.93 to −0.62) and with parental anxiety (rho = 0.79, 95% CI: 0.59 to 0.90). Each association persisted in this sample when the other was included as a covariate along with age (−0.79 and 0.82), but this cannot establish independent effects with 24 observations. None of the measured elbow or shoulder parameters showed a statistically supported association with symptom scores after adjustment for age; forearm rotation was absent on examination in every participant, all of whom had radiographically confirmed complete synostosis, so the motion arc could not differentiate severity between participants. Child and parent reports correlated strongly (rho = 0.80) and differed by 0.1 points on average. No difference between the two aetiological groups was detected on any psychometric measure in this small sample, although the groups differed in total shoulder rotation (182.1° vs. 158.0°, *p* = 0.001, independent of age). Conclusions: In this sample, psychological symptom scores were associated with self-appraised resilience and parental anxiety. No statistically supported age-adjusted association was detected with the measured elbow or shoulder parameters, although the imprecise estimates do not exclude associations of potential clinical interest; because disease-specific functional impairment was not measured, its relationship with psychological symptoms remains unresolved. These exploratory findings support considering psychological assessment alongside functional evaluation, with attention on parental mental health. Disease-specific and task-based measures of functional limitation remain to be examined.

## 1. Introduction

Radioulnar synostosis is a bony or fibrous bridge between the radius and ulna that limits forearm pronation and supination. It arises in two ways. The congenital form follows a failure of segmentation of the proximal forearm in embryogenesis and is the commonest congenital anomaly of the forearm, albeit rare in absolute terms [1,2]. The post-traumatic form complicates roughly 1–6% of both-bone forearm fractures [3,4]. In children, the congenital form is the more frequently seen of the two.

The two forms differ in more than origin. Congenital cases usually present around school age, when tasks requiring forearm rotation begin to matter, and milder cases can go unrecognised for years [1,5]. Bilateral involvement is common and often familial, and associations with sex chromosome aneuploidies and with Apert, Poland and Holt-Oram syndromes are described [1]. Post-traumatic cases are almost always unilateral and follow a single injury in a child whose motor development is already established. The same anatomical endpoint is therefore reached by two quite different developmental routes: a limitation present from birth, or one acquired after normal function.

Functional outcome has been studied in some depth. The largest paediatric outcomes series, a three-centre European cohort of 97 patients, used the Mayo Elbow Performance Score and the QuickDASH [6]; other series have used the ABILHAND-Kids scale [7] or the Jebsen–Taylor test [8]. A recent review notes that the literature consists largely of case reports and small series [1].

That an upper-limb difference has psychosocial consequences for a child and their family is not in serious doubt, and we did not set out to establish it. The open question is a different one. Children with radioulnar synostosis are followed in orthopaedic clinics, where the outcome measures in routine use are functional ones, and where the implicit assumption is that a child whose arm works better is doing better. Whether psychological symptoms actually track the measures that clinicians record has not been tested. The answer bears directly on how these children are followed: if functional status is a poor guide to psychological wellbeing, screening cannot rest on it.

The psychosocial literature on this condition is thin and almost entirely indirect. Case reports describe effects on self-esteem and mention depression, anxiety, aggressive behaviour and difficulty with peers as the child grows older, particularly when the deformity is visible [5]—claims that traced back to other case reports rather than to systematic assessment. One cohort study of 92 children with congenital radioulnar synostosis applied a validated quality-of-life instrument, the PedsQL, and found an inverse association between symptom severity and quality of life [9]; it used a generic quality-of-life measure rather than a psychiatric symptom instrument, and reported neither parental mental health nor a comparison between informants. We are not aware of any study that has measured anxiety and depressive symptoms of this condition with a standardised symptom instrument, collected reports from more than one informant, assessed parental mental health, or tested whether symptom scores relate to the measures of limb function recorded at follow-up.

The gap is not peculiar to radioulnar synostosis. A survey of 107 clinicians working with congenital hand and upper limb differences found that 38% routinely screen for mental health concerns and 12% use standardised outcome measures [10]. Where screening does not happen, the functional score is what the clinician has.

We therefore assessed children with unilateral, non-syndromic radioulnar synostosis at a single visit, performing the orthopaedic examination and the psychometric assessment together. The study was exploratory throughout and was designed to generate hypotheses rather than to test them. We set out to describe symptom scores, quality of life and self-appraised resilience using standardised instruments; to see whether these relate to the measures of elbow and shoulder function recorded in this clinic; to compare congenital and post-traumatic cases; and to examine how closely child and parent reports correspond.

## 2. Materials and Methods

### 2.1. Design and Setting

This was a cross-sectional assessment of a consecutive clinical sample identified retrospectively. Cases were traced through the records of the Department of Orthopaedics and Traumatology, Baltalimanı Bone Diseases Training and Research Hospital, Istanbul, and potentially eligible patients were invited to a single study visit where the orthopaedic examination and all psychometric measures were completed. Final eligibility was confirmed during this visit. Orthopaedic and psychometric data are therefore contemporaneous for every participant.

The study was approved by the Institutional Review Board of Baltalimanı Bone Diseases Training and Research Hospital (approval 38/292, 4 July 2025) and conducted in accordance with the Declaration of Helsinki. Participants under 18 years gave written assent and their parent or guardian gave written informed consent.

### 2.2. Participants

Records were screened for patients treated for radioulnar synostosis between 2015 and 2024. Inclusion criteria were radiologically confirmed radioulnar synostosis of either aetiology, age 9–18 years at assessment, unilateral involvement, and no recognised syndromic diagnosis. Bilateral and syndromic cases were excluded so that the two aetiological groups would be comparable in laterality and free of the distinct developmental profile that accompanies syndromic presentations. The age range corresponds approximately to that in which the child self-report instruments have been used and validated, although the Turkish RCADS validation sample extended only to 17 years and no Turkish paediatric validation of the CD-RISC-10 exists (Section 2.3).

**Figure 1:**
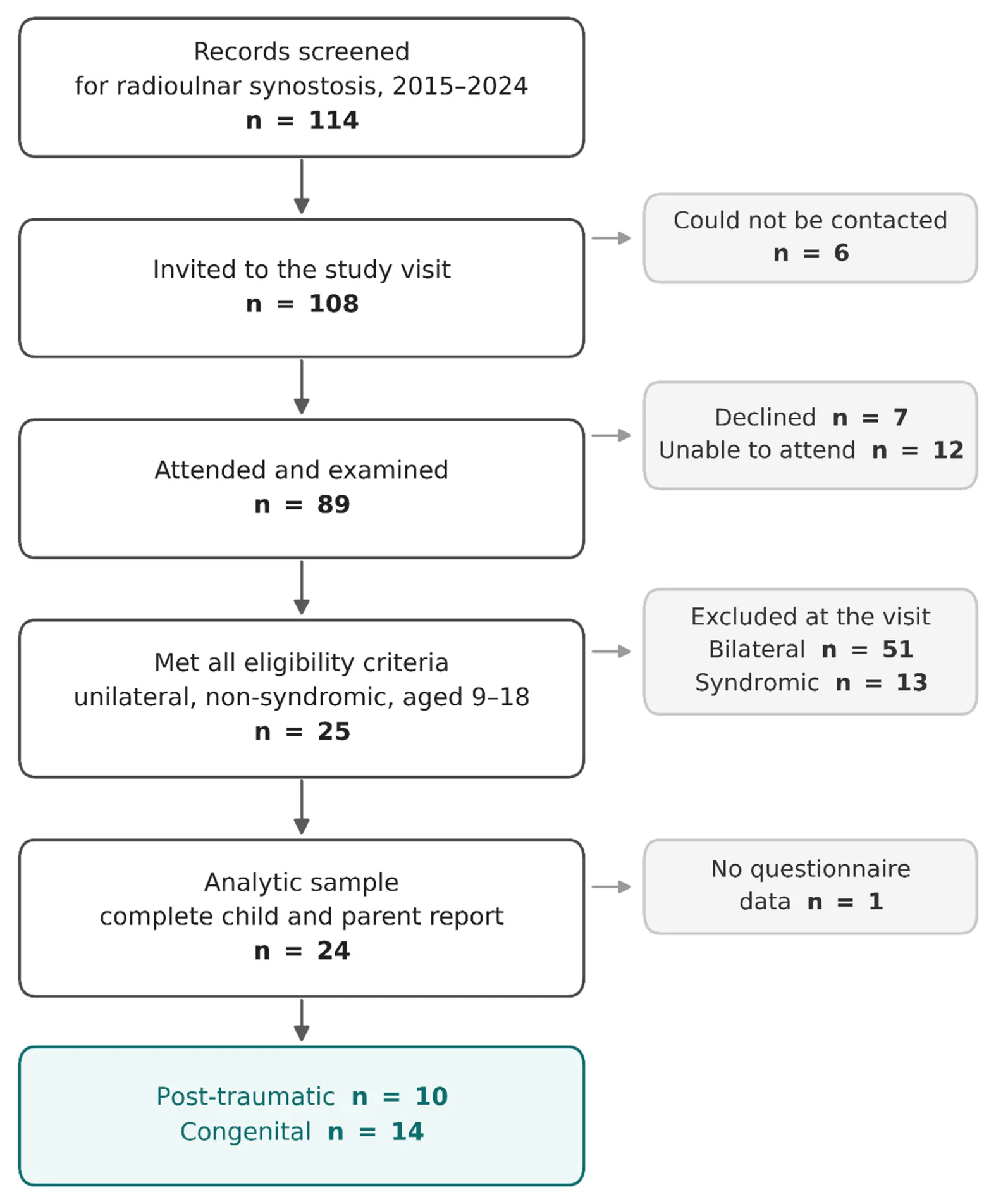
*Participant flow.* Records were screened for patients treated for radioulnar synostosis between 2015 and 2024. Eligibility for unilateral involvement and absence of a syndromic diagnosis was confirmed at the study visit rather than from the records, which is why most exclusions occur after attendance; exclusion at the visit is not equivalent to non-attendance. It is not known how many of the nineteen invited non-attenders would have met the final eligibility criteria.

A total of 114 patients were identified; 6 could not be reached, leaving 108 who were invited. Seven families declined and twelve were unable to attend. Eighty-nine patients attended and were examined. Fifty-one were excluded for bilateral involvement and thirteen for a syndromic diagnosis, leaving twenty-five who met all the criteria. One of these completed no questionnaires, so twenty-four formed the analytic sample, all involving both a child and parent report. The full flow is shown in Figure 1. Eligibility for unilateral involvement and the absence of a syndromic diagnosis was confirmed at the study visit rather than from the records, which is why most exclusions occur after attendance; exclusion at the visit is not equivalent to non-attendance. It is not known how many of the nineteen invited non-attenders would have met the final eligibility criteria.

### 2.3. Measures

#### 2.3.1. Child Self-Report

RCADS-25. Twenty-five items scored 0 (never) to 3 (always), giving a Total Anxiety subscale (15 items, 0–45), a Total Depression subscale (10 items, 0–30) and a total score (0–75); higher scores mean more symptoms. We used the official Turkish youth and caregiver versions distributed by the RCADS website [11]. Both derive from the Turkish 47-item RCADS, validated in clinical samples of 483 children aged 8–17: the child version gave a total alpha of 0.95 with subscales from 0.75 to 0.86 [12], and the parent version showed good internal consistency, test–retest reliability and convergent, divergent and discriminant validity, with a factor structure matching the original six DSM-related factors [13]. The 25 items are a subset of the 47; the Turkish short forms have not been separately validated. The RCADS website distributes both a set of normative tables derived from United States and Icelandic samples and a scoring programmeme (v07.0006) containing 28 normative sets in nine languages; none are Turkish, and no Turkish normative data exist for any version of the instrument.

KIDSCREEN-10 Index. Ten items on a 5-point scale. The Turkish child and proxy versions were validated in 662 children aged 8–18 and 552 parents, with alphas of 0.69–0.95 and 0.68–0.94 across the KIDSCREEN-52, KIDSCREEN-27 and KIDSCREEN-10 forms [14]. Items 3 and 4 are negatively worded and were reversed before summing; the supplementary general health item is not part of the index and was analysed on its own. Raw sums (10–50) were converted to Rasch person parameters and then to T-values using the official international tables, which derive from a twelve-country European sample and are scaled to a mean of 50 and standard deviation of 10 [15]. Separate tables were used for the self-report and proxy forms. Both raw and T-scores are reported; higher values mean better quality of life.

CD-RISC-10. Ten items scored 0 (not true at all) to 4 (true nearly all the time), total 0–40, with higher scores indicating greater self-appraised resilience. The Turkish 25-item CD-RISC was validated in adult trauma survivors [16], and a Turkish version of the 10-item form has been published [17]. Neither has been validated in Turkish children or adolescents, so we examined internal consistency directly in this sample.

#### 2.3.2. Parent Report

RCADS-25-CG, the caregiver counterpart of the RCADS-25, identical in items, response format, subscales and scoring [18].

KIDSCREEN-10 proxy version, scored as the child form [14].

Global health. A single item rating the child’s overall health from 1 (poor) to 5 (excellent).

DASS-21. Parents completed the Turkish DASS-21 [19,20] about their own symptoms over the preceding week. Items score 0–3; subscale sums were doubled, as the manual specifies, giving a range of 0–42 per subscale. No Turkish severity thresholds have been published, so the original cut-offs were used.

#### 2.3.3. Orthopaedic Assessment

Each participant was examined once by each of two orthopaedic surgeons, who took their measurements independently. The mean of the two was used in the analyses. Inter-rater reliability was assessed with a two-way random-effects model for absolute agreement. ICC(2,1) is reported to describe the reliability of an individual examiner’s measurement, followed by ICC(2,2), because the mean of the two measurements was the value analysed. Reliability was computed for every continuous orthopaedic measure with sufficient between-participant variability; shoulder flexion and abduction were at the same value in 22 of 24 participants and were therefore not included. Values are given in Section 3.5 and Table S5.

The orthopaedic examiners were unaware of the participants’ psychometric scores at the time of examination, and questionnaire scoring was carried out independently of the orthopaedic measurements. Blinding to aetiology was not feasible, since aetiology was evident from the clinical history and, in some cases, from previous treatment.

Active range of motion was measured with a goniometer and recorded in degrees for elbow flexion, elbow extension deficit, shoulder flexion, shoulder abduction, shoulder external rotation and total shoulder rotation, the last being the arc from maximum internal to maximum external rotation. Shoulder rotation was measured with the patient seated, the shoulder abducted to 90° and the elbow flexed to 90°, with the scapula stabilised manually. All values are for the affected limb, recorded to the nearest 5°. The fixed forearm position was classified during clinical examination using a 20° criterion: a forearm resting between 20° of pronation and 20° of supination was recorded as neutral, one beyond 20° of pronation as fixed in pronation, and one beyond 20° of supination as fixed in supination. The classification was made clinically rather than radiographically, and only the resulting category, not the underlying angle, was recorded in the dataset. Elbow function was scored with the Mayo Elbow Performance Score (MEPS, 0–100), which combines pain, motion arc, stability and activities of daily living.

Forearm rotation was assessed and documented for every participant as part of the standard clinical examination, and the absence of true radioulnar pronation–supination motion was recorded in each participant’s examination record at the study visit. This information was recorded contemporaneously on the original study-visit examination forms and was not reconstructed retrospectively. Complete synostosis was confirmed radiographically in all participants. Absence of rotation was recorded as a clinical finding rather than being quantified with a goniometer, since there was no arc to measure. Because the variable showed no between-participant variability, it could not serve as a continuous measure of severity or be entered into correlational analyses. What varied clinically was the fixed rotational position of the forearm, recorded as supinated, neutral or pronated. The MEPS, although it includes a motion-arc component, is not a disease-specific measure for radioulnar synostosis and does not quantify either fixed rotational position or the functional consequences of absent forearm rotation. The implications of this for what the orthopaedic variables can and cannot represent are addressed in Section 4.1 and Section 4.7.

### 2.4. Data Handling

Questionnaires were administered through Qualtrics XM Platform and scored according to each manual, with negatively worded items recoded before summing. Subscale and total scores were computed only where all items were present, with one exception: one participant had a single missing CD-RISC-10 item, and the total was prorated from the remaining nine using that participant’s mean. Apart from this, the dataset was complete. No multiple or model-based imputation was performed.

The dataset was defined before the study visit and comprised the variables recorded in routine orthopaedic follow-up together with the psychometric battery. Available clinical variables were elbow and shoulder range of motion, MEPS, fixed forearm position, number of surgical procedures, affected side, age at diagnosis, and time since diagnosis. Family variables were the responding parent’s sex, maternal and paternal education (available for 19 of 24 families) and paternal age. Several variables that might bear on either adaptation or psychological outcome were not collected systematically: hand dominance, perceived visibility of the deformity, detailed injury-severity measures, task-specific functional difficulty, socioeconomic circumstances, family psychiatric history, and any current or previous psychiatric diagnosis or treatment in the child. Pain was not assessed separately; it contributes to the MEPS, but the MEPS is not a substitute for a dedicated pain measure. Age at injury was not available consistently for the post-traumatic participants, so age at diagnosis was used as the single time origin applicable to both groups.

Raw RCADS scores were used throughout, since no Turkish normative data exist. For the sensitivity analysis in Section 3.6, raw scores were also converted to T-scores using the official RCADS scoring programme (v07.0006) [18,21,22]. United States norms were applied, matched on sex and on grade band (3–4, 5–6, 7–8, 9–10, 11–12), with grade derived from age as age minus six in accordance with the programme’s instructions. Separate tables were used for the youth and caregiver forms. The programme holds 28 normative sets in nine languages, none Turkish; the T-scores serve only to test whether the observed age associations persist after age-norming, and they support no normative claim [18,21].

### 2.5. Statistical Analysis

Analyses were run in IBM SPSS Statistics v25 (IBM Corp., Armonk, NY, USA). Cliff’s delta with bootstrap confidence intervals, the Benjamini–Hochberg correction, and the leave-one-out analysis are not available in SPSS and were computed in Python 3.11 (Python Software Foundation, Beaverton, OR, USA) with NumPy 2.4, pandas 3.0 and SciPy 1.17. Rank-based methods were used for all group comparisons, correlations and one-sample comparisons, given the sample size and non-normal distributions. Tests were two-sided at alpha = 0.05.

Continuous variables are given as mean ± SD and median (IQR), while categorical variables are given as counts and percentages. Groups were compared with the Mann–Whitney U test and Fisher’s exact test, with Cliff’s delta and 95% percentile bootstrap intervals (5000 resamples) as effect sizes. Differences within child–parent dyads were tested with the Wilcoxon signed-rank test. The KIDSCREEN-10 T-score distributions were compared with the international reference mean of 50 using the one-sample Wilcoxon signed-rank test, with the Hodges–Lehmann estimate and its bootstrap confidence interval reported alongside; the one-sample *t* test is given as a check. Associations are Spearman correlations; adjusted associations are partial rank correlations, computed by residualising both variables on the rank-transformed covariates. Given the sample size, we fitted no multivariable regression models. Internal consistency was assessed with Cronbach’s alpha.

All analyses in this study are exploratory. The eligibility criteria and the measurement battery were fixed before any participant was assessed, but no analysis plan was registered in advance, no hypothesis was designated as primary before the data were examined, and no result should be read as a confirmatory test. A complete inventory of every analysis reported, in the main text and in the supplement, is given in Table S6. Three associations nonetheless correspond to the questions around which the study was designed and are treated as its principal exploratory analyses: child-reported symptom scores against parental psychological symptoms, against the child’s self-appraised resilience, and against the measured elbow and shoulder parameters. The Section 4 and Section 5 rest on these three. Everything else reported here—the aetiological comparisons, the child–parent concordance analyses, the exploratory subgroup analysis of shoulder rotation, and the sensitivity analyses—is secondary, reported for completeness and for transparency about the number of analyses performed rather than as a basis for inference.

Because many associations were examined, Benjamini–Hochberg adjusted values are given alongside unadjusted *p* values. Tests were grouped by the domain of the predictor rather than by the outcome: for the group comparisons, child self-report (5 tests), parent report of the child (5 tests) and parental self-report (3 tests); for the correlations, parental mental health (4 tests), orthopaedic function (6 tests) and clinical or other variables (4 tests); and, separately, the exploratory subgroup analysis of shoulder rotation against five psychometric outcomes. These groupings were made when the analyses were assembled rather than before data collection, and we note that dividing tests into domains yields a less severe correction than a single family would. Both adjusted and unadjusted values are therefore reported, and interpretation rests on the estimates and their confidence intervals rather than on whether a threshold was crossed.

Confidence intervals for correlations, partial correlations, Cliff’s delta and between-group location differences were obtained by percentile bootstrap with 5000 resamples, with the random seed fixed for reproducibility. For group comparisons, resampling was stratified by aetiology so that each resample retained 10 post-traumatic and 14 congenital participants. Ties were handled by the standard mid-rank method throughout, and resamples in which a variable became constant were discarded. Between-group differences are additionally summarised as Hodges–Lehmann location shifts. For each partial correlation, the number of covariates and the degrees of freedom for the test are reported. Partial rank correlations were computed by rank-transforming both variables and all covariates, residualising each variable on the covariates by ordinary least squares, and correlating the residuals. For correlations and partial correlations, participant pairs were resampled without stratification; for group comparisons and Cliff’s delta, resampling was stratified by aetiology. Within each resample for a partial correlation, the rank transformation and the residualisation were recomputed from the resampled data. The random seed was fixed at 7.

Shoulder flexion and abduction were measured but not entered into the correlational analyses: both sat at the maximum recorded value in 22 of 24 participants, leaving too little variance for a correlation to be meaningful.

One exploratory analysis was confined to the congenital subgroup, in which total shoulder rotation was examined against five psychometric outcomes. These *p* values were adjusted as a separate conceptual family, and the association was probed further with age adjustment, additional adjustment for MEPS, and leave-one-out recomputation. The *p* values for correlations and partial correlations are those of the corresponding rank statistic, obtained from the *t* approximation with the degrees of freedom stated. The confidence intervals come from the bootstrap. The two procedures can diverge in a sample of this size, and where they do, we regard the interval as the more informative summary.

Since the two aetiological groups differed in several clinical respects, comparisons and correlations were repeated with age as a covariate. Age-adjusted group comparisons regressed the rank-transformed outcome on group and rank-transformed age and tested the group term. For the correlations, both unadjusted and age-adjusted estimates are tabulated. For the psychometric group comparisons, age adjustment altered the significance of one of thirteen comparisons (DASS-21 stress, *p* = 0.067 unadjusted, 0.047 adjusted; *q* = 0.142 after correction within its family). Unadjusted values are tabulated and age-adjusted results are given in the text where they differ.

No sample size calculation was performed. The sample was set by the number of eligible consecutive patients with this rare condition at one centre.

## 3. Results

### 3.1. Sample

Of the twenty-five eligible patients who attended, twenty-four completed the psychometric assessment: ten post-traumatic and fourteen congenital, all aged 9–17. Parent questionnaires were completed by the mother in 17 cases (71%) and the father in 7 (29%). Participant flow is shown in Figure 1.

The groups were similar in age at assessment (13.5 ± 3.7 vs. 12.2 ± 2.7 years, *p* = 0.271), sex (female 40% vs. 43%, *p* = 1.000) and affected side (right 50% vs. 57%, *p* = 1.000). Age at diagnosis differed sharply, with almost no overlap between distributions (8.9 ± 1.8 vs. 4.0 ± 1.8 years, *p* < 0.001; ranges 6–12 and 1–7), and time since diagnosis was correspondingly shorter in the post-traumatic group (4.6 ± 3.1 vs. 8.2 ± 3.4 years, *p* = 0.040). Because the pronation–supination arc was uniformly absent, the fixed rotational position of the forearm is the informative descriptor of forearm orientation in this cohort. The forearm was neutral in 21 participants (11 congenital, 10 post-traumatic) and fixed in pronation in three (all congenital); no participant was fixed in supination (*p* = 0.239). A family history of the condition was likewise present in three congenital patients only (*p* = 0.239). Operative treatment followed the natural history of the two forms: five of ten post-traumatic patients had undergone at least one procedure against one of fourteen congenital patients (*p* = 0.050), with means of 1.1 ± 1.2 and 0.1 ± 0.3 procedures (*p* = 0.013). Table 1 gives the full characteristics.

### 3.2. Psychometric Findings

Internal consistency was acceptable to good throughout (Table S3): RCADS-25: child 0.859, Total Anxiety: 0.747, Total Depression: 0.770; KIDSCREEN-10 child: 0.810; CD-RISC-10: 0.896; RCADS-25-CG: 0.830; KIDSCREEN-10 proxy: 0.861; DASS-21 total: 0.888, with subscale alphas of 0.828 (depression), 0.723 (anxiety) and 0.705 (stress).

Across the sample, mean scores were 20.0 ± 8.3 on the RCADS-25 (range 6–32), 35.1 ± 6.1 on KIDSCREEN-10 (25–44), 20.5 ± 7.3 on CD-RISC-10 (8–36) and 20.1 ± 7.7 on RCADS-25-CG (7–34). Parental distress was modest on average (DASS-21 depression 8.8 ± 7.4, anxiety 8.5 ± 5.9, stress 11.2 ± 5.8). Converted to the international KIDSCREEN metric, quality of life fell below the reference mean of 50 on both forms: child self-report averaged 43.1 (95% bootstrap CI: 40.5 to 45.6) and parent proxy report averaged 41.2 (37.2 to 45.5), approximately 0.7 and 0.9 reference standard deviations below the norm. Because the child T-scores departed from normality (Shapiro–Wilk W = 0.89, *p* = 0.013), each distribution was compared with the reference mean using the one-sample Wilcoxon signed-rank test: child self-report *p* < 0.001, Hodges–Lehmann estimate 43.5 (95% CI: 40.4 to 46.4); parent proxy report *p* = 0.002, Hodges–Lehmann estimate 40.8 (36.3 to 45.4). A one-sample t test gives the same conclusion on both forms (*p* < 0.001). This was the only measure for which an international reference metric could be applied; RCADS scores were not interpreted normatively. The KIDSCREEN reference sample was drawn from twelve European countries and differs from the present sample culturally and in period, and no local control group was available, so the difference cannot be attributed to radioulnar synostosis.

No psychometric difference between the aetiological groups was detected in this sample (Table 2). Estimated between-group differences, expressed as Hodges–Lehmann location shifts, are given with bootstrap confidence intervals in Table 2; all intervals included zero. For the RCADS-25 total, the shift was −4.0 points (95% CI: −12.0 to +5.0), an interval wide enough to be compatible with a clinically meaningful difference in either direction. The absence of a detected difference should therefore not be read as evidence that the two aetiologies have similar psychological profiles. The estimates were imprecise and every confidence interval crossed zero: RCADS-25 total 17.8 ± 9.3 vs. 21.6 ± 7.5 (*p* = 0.241, *q* = 0.603, Cliff’s δ = −0.29, 95% CI: −0.74 to 0.23); Total Anxiety (*p* = 0.197, *q* = 0.603); KIDSCREEN-10 35.2 ± 6.4 vs. 35.1 ± 6.1 (*p* = 0.860); CD-RISC-10 (*p* = 0.519); RCADS-25-CG (*p* = 0.291, *q* = 0.484); and the parental rating of the child’s global health was lower in the congenital group but not significantly so (3.4 ± 1.2 vs. 2.6 ± 0.9, *p* = 0.092, *q* = 0.461, δ = 0.40). Parents’ own symptoms did not differ (all *q* ≥ 0.200), though DASS-21 stress was somewhat higher in the post-traumatic group (13.6 ± 6.0 vs. 9.6 ± 5.2, *p* = 0.067, *q* = 0.200, δ = 0.45). Age adjustment did not materially change these comparisons; for parental stress, the age-adjusted *p* value was 0.047, with *q* = 0.142 after correction within the parental self-report family.

### 3.3. What Symptoms Track

Correlations were grouped into three conceptual families (Table 3).

**Figure 2:**
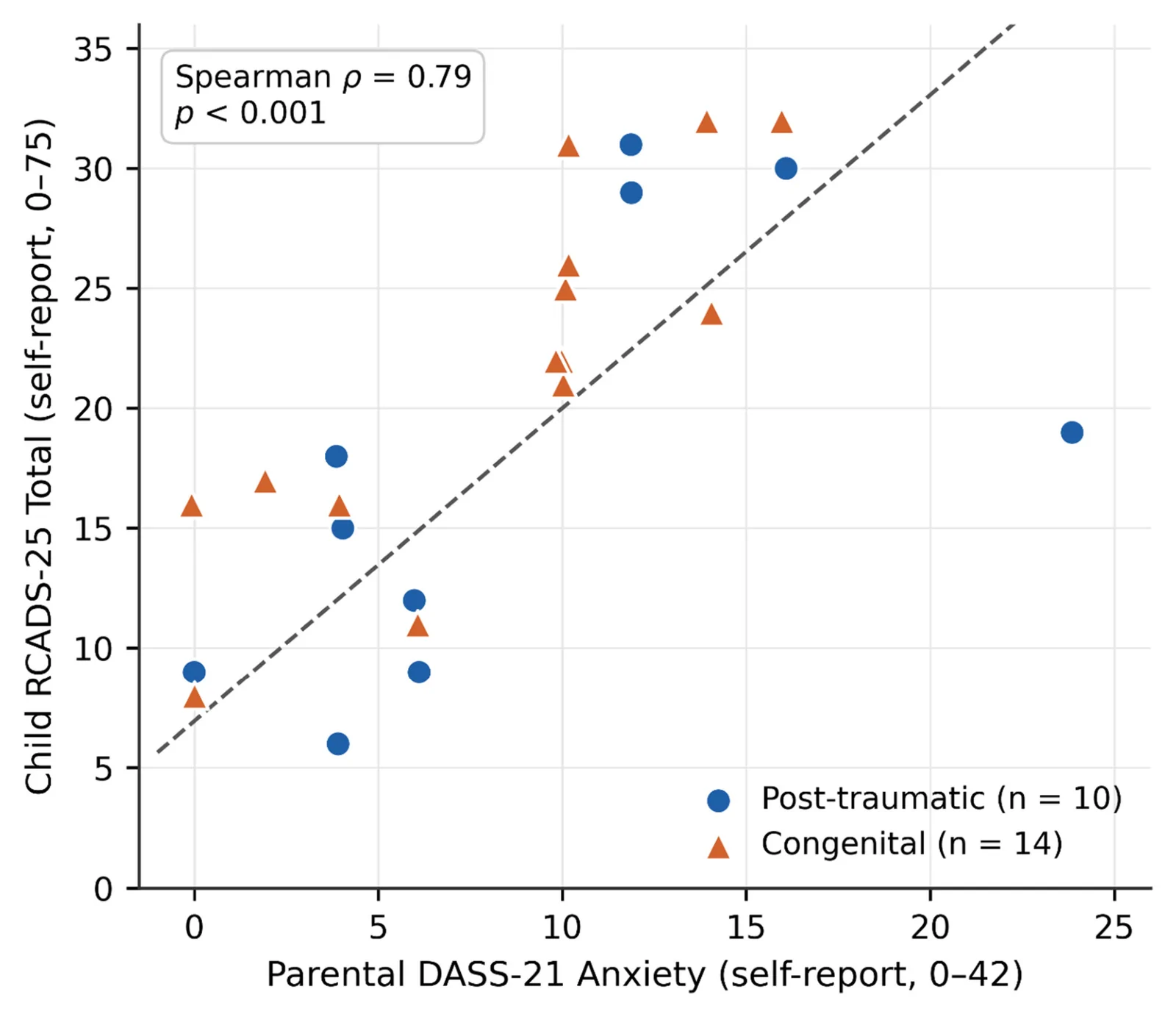
*Parental anxiety and child-reported symptoms (n = 24).* Each point is one child–parent dyad. Parental DASS-21 Anxiety is parent self-report and child RCADS-25 Total is child self-report, so the association is cross-informant and cannot readily be explained by same-informant method variance. Circles denote post-traumatic cases (n = 10); triangles denote congenital cases (n = 14). The dashed line is a Theil–Sen robust fit shown for orientation; the reported statistic is Spearman’s ρ. A small horizontal jitter (±0.18) separates coincident points. Adjustment for age and aetiology left the association essentially unchanged (partial ρ = 0.80, *p* < 0.001).

Parental mental health. Parental anxiety was strongly associated with the child’s own reported symptoms (rho = 0.79, 95% CI: 0.59 to 0.90, *p* < 0.001; age-adjusted 0.78, 95% CI: 0.54 to 0.89, *q* < 0.001), as was the DASS-21 total score (0.68, 95% CI: 0.37 to 0.85; age-adjusted 0.69, 95% CI: 0.34 to 0.85, *q* = 0.001). The associations with the depression and stress subscales were weaker and less certain: the age-adjusted estimates were 0.41 and 0.43 (*q* = 0.049 for both), but both were imprecisely estimated, and the bootstrap interval for depression extended from −0.15 to 0.85. Among the parental measures, only anxiety and the total score were estimated with any precision. DASS-21 was completed by parents about themselves and the RCADS-25 by children about themselves, so these are cross-informant associations that cannot readily be explained by same-informant method variance. Figure 2 shows the association with parental anxiety.

Orthopaedic function. None of the orthopaedic variables showed a statistically supported association with child-reported symptoms after adjustment for age. MEPS showed an unadjusted association (rho = −0.47, *p* = 0.021) that fell away after adjustment (−0.36, *p* = 0.091, *q* = 0.499). Number of surgical procedures (age-adjusted −0.11, *q* = 0.606), total shoulder rotation (−0.14, *q* = 0.606), shoulder external rotation (−0.22), elbow flexion (0.21) and elbow extension deficit (0.22) likewise showed no statistically supported age-adjusted association (*q* = 0.499 for the last three).

Clinical and other variables. Symptoms were inversely associated with self-appraised resilience (rho = −0.84, 95% CI: −0.93 to −0.62; age-adjusted −0.74, 95% CI: −0.90 to −0.37, *q* < 0.001, Figure S3) and with quality of life (−0.60; age-adjusted −0.42, *q* = 0.091). Neither age at diagnosis (−0.30, *q* = 0.168) nor time since diagnosis (0.35, *q* = 0.132) was associated after adjustment.

Resilience and parental anxiety were themselves inversely correlated (rho = −0.47, 95% CI: −0.79 to −0.05, *p* = 0.020), so they are not independent of one another. Each nevertheless retained its association with symptom scores in this sample when the other was included as a covariate: with age and parental anxiety held constant, resilience remained associated with symptoms (partial rho = −0.79, 95% CI: −0.92 to −0.52, *p* < 0.001, 2 covariates, 20 degrees of freedom for the partial-correlation test); with age and resilience held constant, parental anxiety remained associated (0.82, 95% CI: 0.45 to 0.92, *p* < 0.001, 2 covariates, 20 degrees of freedom for the partial-correlation test). This is a within-sample statistical observation and does not establish that either variable has an independent causal or predictive effect; with 24 observations, cross-sectional data and overlapping constructs, such a conclusion would not be warranted. Resilience was also associated with parent-reported symptoms (−0.66, 95% CI: −0.89 to −0.28; age-adjusted −0.55, *p* = 0.006), so the association is not confined to the informant who supplied both measures.

### 3.4. Sensitivity Analyses for the Parental Anxiety Association

The association between parental anxiety and child-reported symptoms was stable under every check applied. It was 0.79 unadjusted (95% CI: 0.59 to 0.90); 0.78 with age controlled (95% CI: 0.54 to 0.89; 1 covariate, 21 degrees of freedom for the partial-correlation test); 0.80 with age and aetiology (95% CI: 0.57 to 0.91; 2 covariates, 20 df); and 0.78 with age, aetiology and MEPS (95% CI: 0.52 to 0.91; 3 covariates, 19 df), all *p* < 0.001. Substituting the age-normed T-score as the outcome gave 0.67 (*p* < 0.001). Recomputing the age-adjusted partial correlation twenty-four times, with each time omitting one participant, gave a range of 0.74 to 0.81, so no single observation drove the result. Leave-one-out analysis addresses the influence of individual observations but not instability arising from sampling variation, residual confounding, nonlinearity, or influential combinations of observations; the bootstrap intervals above give a fuller picture of the precision of these estimates in a sample of this size. These sensitivity analyses assess the stability of the estimate under alternative specifications; they do not convert an exploratory association into a confirmatory finding.

### 3.5. Orthopaedic Findings

Inter-rater reliability was good to excellent across the orthopaedic measures (Table S5). ICC(2,1), describing the reliability of a single examiner’s measurement, was 0.92 for total shoulder rotation, 0.91 for elbow flexion, 0.92 for elbow extension deficit, 1.00 for shoulder external rotation and 0.76 for MEPS. For the mean of the two examiners’ measurements, which was the value analysed, the corresponding ICC(2,2) values were 0.96, 0.95, 0.96, 1.00 and 0.86.

Elbow measures did not separate the groups: elbow flexion: 145.0 ± 14.3° vs. 135.0 ± 19.5° (*p* = 0.168), extension deficit: 4.0 ± 8.4° vs. 3.2 ± 7.2° (*p* = 1.000), shoulder flexion: 180.0 ± 0.0° vs. 172.9 ± 18.2° (*p* = 0.246). MEPS was higher in the post-traumatic group (95.0 ± 4.7 vs. 84.6 ± 7.5, *p* = 0.003), but clustered at the top of the scale there (median 95.0, IQR 95.0–98.8) against a wider spread in the congenital group (median 85.0, IQR 80.0–85.0).

Shoulder rotation was the clearest orthopaedic difference. External rotation (87.0 ± 5.4° vs. 98.6 ± 8.4°, *p* = 0.002) and total rotation (158.0 ± 6.3° vs. 182.1 ± 15.8°, *p* = 0.001) were both greater in the congenital group, with little overlap between distributions (Figure S1). Unlike every psychometric comparison, this difference was independent of age: total shoulder rotation did not correlate with age (rho = −0.19, *p* = 0.377) and the group difference persisted after adjustment (*p* < 0.001).

In an exploratory subgroup analysis confined to the fourteen congenital participants, greater total shoulder rotation went with higher self-appraised resilience (rho = 0.62, *p* = 0.018, *q* = 0.091; Figure S2). The association was one of five examined against shoulder rotation in this subgroup and did not survive correction for multiplicity. Supporting detail, including age and MEPS adjustment and leave-one-out recomputation, is given in the Supplementary Materials. Given the subgroup size and the number of tests, this observation is reported for completeness and should be regarded as hypothesis-generating only.

### 3.6. Child–Parent Concordance and Age Associations

Child and parent reports were strongly correlated, with no systematic group-level difference in score level (Table S2). Agreement at the level of individual dyads was not formally assessed. For the RCADS-25 total, rho was 0.80 (age-adjusted 0.75) with a mean difference of 0.1 points on a 0–75 scale (*p* = 0.735). Agreement was highest for anxiety (0.84; age-adjusted 0.82) and lower for depression (0.64; 0.55). Child and proxy quality of life also correlated (0.69; 0.65).

Child-reported symptoms, resilience and quality of life were all associated with age in raw scores (rho = −0.61, 0.61 and 0.52). A sensitivity analysis standardising RCADS-25 scores against an available non-Turkish reference set is given in Table S1: the age association weakened and lost significance for child self-report but persisted for parent report. Within the post-traumatic group, time since diagnosis was not associated with symptoms once age was accounted for (−0.05, *p* = 0.892); the two were themselves strongly correlated in this subgroup (0.59) and cannot be separated at this sample size.

## 4. Discussion

### 4.1. Measured Function and Symptom Scores Were Not Aligned

As far as we know this is the first study to measure anxiety and depressive symptoms in paediatric radioulnar synostosis with a standardised symptom instrument and reports from more than one informant. What follows is a discussion of each observation in turn, with the caveats that a sample of 24 and an exploratory design impose.

No statistically supported age-adjusted association was detected between symptom scores and any of the orthopaedic variables measured. A child scoring 95 on the MEPS may report as many symptoms as scoring 80. The estimates are imprecise, however, and do not exclude associations of potential clinical interest: the age-adjusted correlation with the MEPS was −0.36 with a 95% confidence interval from −0.64 to 0.02. What can be said is that no supported association was detected in this sample, which is a reason to assess psychological symptoms alongside functional outcome rather than to infer them from it.

This needs a qualification, and it is an important one. Forearm rotation was absent on examination in every participant, all of whom had radiographically confirmed complete synostosis; the motion arc was therefore uniform across the sample and could neither serve as a measure of severity nor enter a correlation. What the analysis therefore shows is that no supported association was detected between symptom scores and the elbow and shoulder parameters recorded at follow-up; it does not show that symptoms are unrelated to disease-specific functional impairment, which was not measured. The MEPS scores pain, motion arc, stability and daily activities, but it is not a disease-specific instrument for this condition: it quantifies neither fixed rotational position nor the functional consequences of absent forearm rotation, and in our sample, it clustered near the ceiling in the post-traumatic group. Its inter-rater reliability was also the lowest of any orthopaedic measure here (ICC(2,1): 0.76 against 0.91–1.00), and a measurement error of that size attenuates any correlation the score might have with the symptoms.

The same absence of a detected association was seen across variables with quite different properties: total shoulder rotation, measured in degrees with a wide spread and no ceiling (age-adjusted −0.14); elbow flexion; and the number of operations undergone (−0.11), which is not a range-of-motion measure at all. None of these is an adequate measure of disease-specific functional severity either, so the pattern does not resolve the question. Because the rotation arc was uniformly absent in this cohort, future work should look to dimensions that can vary between patients: fixed rotational position quantified in degrees, task-based functional performance, compensatory motion, and patient-reported upper-extremity function.

### 4.2. Parental Anxiety

The association between parental anxiety and child-reported symptoms was the strongest and most durable finding here, and its standing rests on the design. Parents completed the DASS-21 about their own symptoms in the preceding week; children completed the RCADS-25 about themselves. The two measures share no items, no respondent and no reference period, so the association cannot readily be explained by same-informant method variance—a common concern when associations rest on measures completed by the same respondent. Other common-source influences, a shared family context or distress in both members of the dyad at the same time, are not excluded by the design.

That parental distress and child internalising symptoms go together is among the better documented findings in child mental health, with pooled estimates of r = 0.23 between maternal depression and child internalising problems across 193 studies [23], an effect size of 0.16 in an earlier meta-analysis [24] and consistent small associations in a later meta-analytic synthesis [25]. The same holds in paediatric chronic illness, where maternal distress predicts poorer child adjustment [26] and caregivers report more parenting stress than caregivers of healthy children (d = 0.40) [27]. Our estimate is numerically larger than these, though the comparison is limited by differences in constructs, measures and samples. Whether it reflects something particular to this population, the small sample, the instruments, or the cross-informant design cannot be settled without a comparison group.

Direction cannot be established from cross-sectional data. Parental distress may contribute to the child’s symptoms, the child’s condition may burden the parent, or both may follow from shared genetic or environmental factors. All three are plausible and none excludes the others.

### 4.3. Resilience

Resilience was the other strong correlate. The two predictors were themselves inversely correlated (−0.47, 95% CI: −0.79 to −0.05), so they are not independent of one another. Each nevertheless retained its association in this sample when the other was included as a covariate: −0.79 for resilience with age and parental anxiety held constant, and 0.82 for parental anxiety with age and resilience held constant. This is a within-sample statistical observation; with 24 observations, cross-sectional data and constructs that overlap in affective valence, it does not establish that either variable acts independently of the other. Age, which was associated with both symptom scores and resilience in unadjusted analysis, did not account for either association. Resilience was also associated with parent-reported symptoms (−0.66, 95% CI: −0.89 to −0.28; −0.55 adjusted for age), so the association is not confined to the informant who supplied both measures.

Inverse associations between resilience and internalising symptoms are well established in children and adolescents, and the direction here is the expected one. Two features of our result deserve comment. The magnitude sits at the upper end of what is usually reported, which in a sample of this size may reflect the wide spread of scores on both measures. And unlike the parental association, both measures came from the child. Same-informant method variance cannot be excluded, as it can for the parental finding, and self-appraised resilience and self-reported anxiety and depression share affective valence at the item level, so some construct overlap is plausible. The two associations are not equivalent in evidential standing, though both are substantial.

### 4.4. Concordance Between Informants

Parent and child reports were strongly correlated, with no systematic group-level difference in score level. Rank correlations of 0.80 for total symptoms and 0.84 for anxiety are high against published benchmarks: a large meta-analysis of the multi-informant approach covering 341 studies reports a pooled parent–child correlation of 0.25 for internalising problems [28], essentially unchanged from the classic estimate of 0.25 for total problems across 119 studies [29]. Agreement on anxiety instruments usually falls between 0.2 and 0.6 [30], and even in neurodevelopmental samples, where agreement runs higher than in community samples, the pooled estimate for internalising problems is 0.42 [31].

No statistically significant paired score difference was detected on any subscale (all *p* > 0.20). In practical terms, parent report may offer useful complementary information when child self-report is unavailable. High concordance does not establish interchangeability, and we make no claim of formal agreement, which would require intraclass correlation or limits-of-agreement analysis. Two further cautions apply: high agreement in a small sample may partly reflect the wide range of symptom scores, and agreement between two reports does not make either accurate. Both could be shaped by the same family-level factors—a possibility our finding on parental distress makes concrete.

### 4.5. Aetiology: Orthopaedic Differences and No Detected Psychometric Difference

Congenital and post-traumatic cases differed substantially in shoulder rotation, whereas no psychometric difference was detected between them in this sample. The estimates were imprecise: the confidence interval for every between-group difference included zero, but the intervals were wide. For the RCADS-25 total, the estimated shift was −4.0 points (95% CI: −12.0 to +5.0). Given the sample size, this should not be read as evidence that the two aetiologies have similar psychological profiles.

Compensatory motion in this condition is not a new idea. A review notes that compensatory motion and hypermobility are often seen at the wrist and shoulder, and that these joints may in consequence be prone to overuse injury [1]. Quantified evidence is sparse but exists: Ogino and Hikino measured compensatory rotation goniometrically in 40 congenital cases, documenting a mean compensatory arc at the wrist from 76.3° of pronation to 42.9° of supination [32]. Experimental work supplies a mechanism for shoulder involvement—when forearm rotation was splinted in healthy adults, three-dimensional kinematics showed the shoulder taking up the restriction, with total ab/adduction increasing by 57.3–62.8° during a task needing pronation and supination [33]. Motion analysis after single-bone-forearm surgery has likewise documented substantial compensatory motion at other joints during daily activities [34].

What we add is the quantification of shoulder rotation in degrees and the observation that it separates the two aetiologies independently of age. We did not demonstrate compensation. Our measurements are of static range of motion at examination, not of joint use during tasks requiring forearm rotation, and we did not measure the contralateral shoulder, so generalised laxity or individual variation in shoulder mobility cannot be distinguished from adaptation specific to the condition. What follows is therefore one plausible interpretation rather than a demonstrated mechanism: in congenital cases, the restriction is present while motor patterns are forming and compensation is built in from the start, whereas in post-traumatic cases, it appears after those patterns are established. One earlier report, available only as a conference abstract, described normal shoulder motion in all 25 congenital patients, with compensatory laxity confined to the wrist [8]. Because detailed methods and degree-based shoulder measurements were not given, this observation should be treated as preliminary and cannot be compared directly with the present measurements; the divergence nonetheless deserves testing in larger samples.

The contrast between the two sets of findings is worth noting. The aetiological groups differed in shoulder mobility, whereas no corresponding psychometric difference was detected in this sample.

An exploratory analysis within the congenital subgroup found greater shoulder rotation associated with higher self-appraised resilience (rho = 0.62, *q* = 0.091); it did not survive correction, rests on fourteen observations, and the apparent absence of the association in the post-traumatic group was not formally tested against the congenital estimate. Details are in Table S7.

### 4.6. Age

Raw symptom scores fell with age while resilience and quality of life rose, which might suggest that younger children with radioulnar synostosis are at greater risk. A sensitivity analysis standardising scores against an available non-Turkish reference set (Table S1) suggests that part of the observed association may reflect age-related scoring patterns in the available reference data: the association weakened and lost significance in child self-report, though it persisted in parent report. We offer no normative interpretation beyond that, as no Turkish reference data exist.

Time since diagnosis was not associated with symptoms in the post-traumatic group once age was accounted for, and the two cannot be separated at this sample size. A cross-sectional design could not in any case distinguish change over time from selection effects, since recently diagnosed children may be more likely to be in active follow-up. That question needs prospective assessment.

### 4.7. Limitations

The sample was small (n = 24), from a single centre, and unequal between groups (10 versus 14). No power calculation was performed; the sample was set by the number of eligible consecutive patients with this rare condition. Every analysis in this study is exploratory; no analysis plan was registered in advance and no result is offered as a confirmatory test. The null aetiological comparisons in particular should not be read as evidence of equivalence.

There was no control group. We cannot say whether children with radioulnar synostosis carry more symptoms than children without it, and we make no normative claim about the RCADS: no Turkish normative data exist, and the official scoring programme holds 28 normative sets in nine languages, none Turkish. All analyses are within-sample. The absence of a control group also means we cannot tell whether the parental association we observed is specific to this condition; testing that would require comparing correlation coefficients between groups in a substantially larger controlled sample.

Throughout this paper, the RCADS-25 results are described as symptom scores, and that is all they are. Raw scores on a screening instrument cannot establish clinically significant anxiety or depression, and in the absence of Turkish normative data and of a validated Turkish short form, we cannot estimate how many participants had clinically important symptoms, let alone a psychiatric disorder. No participant in this study underwent a diagnostic assessment. Statements about elevated or clinically significant symptoms are therefore avoided, and the T-score conversion reported in Table S1 is used only to test whether an age association persisted after age-norming.

Two instruments lack Turkish validation in the relevant population. No validation exists for the Turkish 25-item RCADS in either version, though the 47-item child version from which it derives was validated in a Turkish clinical sample aged 8–17 [12]. The Turkish CD-RISC-10 has not been validated in children or adolescents; internal consistency here was good (alpha = 0.896), but scores in the younger participants should be read with caution.

The design was cross-sectional, so the direction of the parent–child association cannot be determined and age effects cannot be separated from cohort effects.

Forearm rotation was absent on examination in all participants, so the motion arc could not differentiate severity between them. Absence of motion was recorded clinically rather than in degrees; in cohorts where residual rotation is present, the remaining arc should be quantified. The MEPS does not specifically assess fixed rotational position or the functional consequences of absent forearm rotation, so neither it nor the measured ranges of elbow and shoulder motion constitute a disease-specific measure of functional impairment in this condition. Fixed forearm position was classified against a 20° criterion rather than recorded as an angle, so the neutral category spans a 40° range and includes forearms resting in mild pronation; it also showed no variance in the post-traumatic group. This limits comparison with series that report the rotational position in degrees, and it may partly account for the proportion of congenital participants classified as neutral here, since congenital synostosis is more often described as fixed in pronation. Future studies should include disease-specific or task-based measures of functional limitation and quantify fixed rotational position in degrees.

Several variables that might bear on both adaptation and psychological outcome were not collected systematically: hand dominance, perceived visibility of the deformity, detailed injury severity, task-specific functional difficulty, socioeconomic circumstances, family psychiatric history, and any current or previous psychiatric diagnosis or treatment in the child. Pain was not assessed separately; it contributes to the MEPS, but the MEPS is not a substitute for a dedicated pain measure. Maternal and paternal education were available for 19 of 24 families; neither was associated with child symptom scores (rho = 0.34, *p* = 0.156 and rho = 0.34, *p* = 0.152) or with parental anxiety (0.05 and 0.14, both *p* > 0.5). The association between parental anxiety and child symptom scores was 0.79 both before and after including the responding parent’s sex as a covariate. Neither check substitutes for the variables that were not collected. Age at injury was not available consistently for the post-traumatic participants, so time since diagnosis should not be read as equivalent to time since the onset of functional restriction.

Post-traumatic stress symptoms were not assessed. In the post-traumatic group, the synostosis follows an injury, and symptoms attributable to that event rather than to the residual restriction cannot be separated from those measured here. The RCADS-25 covers anxiety and depressive symptoms and does not include a trauma-specific scale. Whether the psychological picture in post-traumatic cases reflects the injury, the restriction, or both is a question this design cannot answer.

Bilateral and syndromic cases were excluded. This improved internal comparability but limits generalisability: bilateral involvement accounts for a substantial proportion of congenital cases—44.1% in a multicentre epidemiological series of 329 patients, larger than any published outcomes cohort, and 60–80% in figures often cited in case reports—and represents the more functionally and cosmetically affected subgroup. Our congenital sample therefore represents a relatively less complex and potentially less severely affected part of the wider population.

Nineteen of the one hundred and eight invited patients did not attend, and the records did not allow them to be compared with attenders on age, sex or aetiology. The relatively high attendance rate reduces, but does not eliminate, concern about selection bias.

### 4.8. Future Directions

Two additions would improve on this work the most.

A control group would settle what the present design cannot. Children attending general paediatric outpatient clinics for routine reasons, frequency-matched on age band and sex, would provide local comparison data for all four instruments—none of which currently has Turkish norms—and would allow the specificity of the parental association to be examined. A second comparison group of children with a healed upper-extremity fracture, at least twelve months from injury with full range of motion and no residual deformity, would separate the effect of permanent functional restriction from that of the injury itself. Future controlled studies, ideally multicentre, should recruit local controls and be powered for the effect sizes reported here. Controls should be selected without screening on the symptom level, since excluding symptomatic children would produce an artificially healthy comparison group.

A prospective follow-up of this sample would address what the cross-sectional design leaves open: the direction of the association between parental distress and child symptoms. Whether psychological symptoms change over time after diagnosis, and whether shoulder rotation develops over time in post-traumatic cases, are secondary longitudinal questions. In cohorts with complete synostosis, future studies should quantify fixed forearm rotational position in degrees and add disease-specific or task-based measures of functional impairment and compensatory motion; where residual forearm rotation is present, the remaining arc should also be quantified. Laterality and syndromic status should be recorded prospectively.

## 5. Conclusions

In children and adolescents with radioulnar synostosis, none of the measured elbow or shoulder parameters showed a statistically supported association with psychological symptom scores. Symptom scores were instead associated with two variables—the child’s self-appraised resilience and the parent’s self-reported anxiety—each of which retained its association in this sample when the other was included as a covariate. Because forearm rotation was absent on examination in every participant, the motion arc was uniform across the sample and could not be examined as a measure of severity; the relationship between symptom scores and disease-specific functional impairment therefore remains open. Quality of life fell below the international reference mean, although without a local control group this cannot be attributed to the condition. No psychometric difference between the aetiological groups was detected in this small sample, although they differed substantially in shoulder rotation, and child and parent reports were strongly correlated with no systematic difference in level. No supported association was detected between symptom scores and the functional measures available at follow-up, although the estimates are imprecise and do not exclude associations of clinical interest; parental mental health warrants attention alongside them. All findings are exploratory and need confirmation in a controlled study.

## Figures and Tables

**Table 1:** Sample characteristics by aetiology.

Variable	Post-Traumatic (n = 10)	Congenital (n = 14)	Difference (95% CI)	*p*
Age at assessment, years	13.5 ± 3.7	12.2 ± 2.7	+1.0 (−1.0 to +5.0)	0.271
—median (IQR)	15.5 (9.2–16.8)	12.0 (10.0–15.0)		
Age at diagnosis, years	8.9 ± 1.8	4.0 ± 1.8	+5.0 (+3.0 to +6.0)	<0.001
—median (IQR)	9.0 (8.0–9.8)	4.0 (2.2–5.0)		
Time since diagnosis, years	4.6 ± 3.1	8.2 ± 3.4	−4.0 (−6.0 to 0.0)	0.040
—median (IQR)	5.5 (1.5–7.0)	8.0 (6.0–11.5)		
MEPS (0–100)	95.0 ± 4.7	84.6 ± 7.5	+10.0 (+5.0 to +15.0)	0.003
—median (IQR)	95.0 (95.0–98.8)	85.0 (80.0–85.0)		
Number of surgical procedures	1.1 ± 1.2	0.1 ± 0.3	+0.5 (0.0 to +2.0)	0.013
—median (IQR)	1.0 (0.0–2.0)	0.0 (0.0–0.0)		
Elbow flexion, °	145.0 ± 14.3	135.0 ± 19.5	+10.0 (0.0 to +20.0)	0.168
Elbow extension deficit, °	4.0 ± 8.4	3.2 ± 7.2	0.0 (0.0 to 0.0)	1.000
Shoulder flexion, °	180.0 ± 0.0	172.9 ± 18.2	0.0 (0.0 to 0.0)	0.246
Shoulder external rotation, °	87.0 ± 5.4	98.6 ± 8.4	−10.0 (−20.0 to −5.0)	0.002
Total shoulder rotation, °	158.0 ± 6.3	182.1 ± 15.8	−30.0 (−40.0 to −10.0)	0.001
Female sex, n (%)	4 (40)	6 (43)		1.000
Right side affected, n (%)	5 (50)	8 (57)		1.000
Fixed forearm position, n (%)				0.239
—neutral	10 (100)	11 (79)		
—pronated	0 (0)	3 (21)		
—supinated	0 (0)	0 (0)		
Family history, n (%)	0 (0)	3 (21)		0.239
≥1 surgical procedure, n (%)	5 (50)	1 (7)		0.050

Mean ± SD unless stated. Mann–Whitney U for continuous variables, Fisher’s exact test for categorical variables. Difference: Hodges–Lehmann location shift (post-traumatic minus congenital), the median of all pairwise differences, with 95% percentile bootstrap confidence interval from 5000 resamples stratified by aetiology. All participants had complete synostosis with no residual forearm rotation, so fixed rotational position rather than motion arc is reported. MEPS, Mayo Elbow Performance Score.

**Table 2:** Psychometric measures by aetiology.

Measure (Range)	Post-Traumatic (n = 10)	Congenital (n = 14)	Difference (95% CI)	*p*	*q*	δ (95% CI)
**Child self-report**						
RCADS-25 total (0–75)	17.8 ± 9.3	21.6 ± 7.5	−4.0 (−12.0 to +5.0)	0.241	0.603	−0.29 (−0.74, 0.23)
—Total Anxiety (0–45)	10.4 ± 5.9	13.5 ± 3.9	−3.0 (−8.0 to +2.0)	0.197	0.603	−0.32 (−0.76, 0.21)
—Total Depression (0–30)	7.4 ± 4.2	8.1 ± 3.8	−1.0 (−4.0 to +3.0)	0.638	0.797	−0.12 (−0.59, 0.38)
KIDSCREEN-10 (10–50)	35.2 ± 6.4	35.1 ± 6.1	+0.5 (−5.5 to +4.0)	0.860	0.860	0.05 (−0.48, 0.54)
CD-RISC-10 (0–40)	21.7 ± 9.2	19.6 ± 5.8	+3.0 (−6.0 to +9.0)	0.519	0.797	0.16 (−0.39, 0.66)
**Parent report of child**						
RCADS-25-CG total (0–75)	18.5 ± 9.9	21.3 ± 5.6	−4.0 (−12.0 to +7.0)	0.291	0.484	−0.26 (−0.76, 0.30)
—CG anxiety (0–45)	10.2 ± 5.5	12.9 ± 3.8	−3.5 (−8.0 to +2.0)	0.238	0.484	−0.29 (−0.77, 0.24)
—CG depression (0–30)	8.3 ± 5.1	8.4 ± 2.6	−1.0 (−3.0 to +5.0)	0.722	0.792	−0.09 (−0.61, 0.44)
KIDSCREEN-10 proxy (10–50)	33.8 ± 6.5	35.3 ± 6.9	−1.0 (−8.0 to +4.0)	0.792	0.792	−0.07 (−0.58, 0.41)
Global health perception (1–5)	3.4 ± 1.2	2.6 ± 0.9	+1.0 (0.0 to +2.0)	0.092	0.461	0.40 (−0.05, 0.79)
**Parental self-report (DASS-21)**						
Depression (0–42)	11.2 ± 8.9	7.1 ± 5.9	+3.0 (−2.0 to +12.0)	0.408	0.612	0.21 (−0.29, 0.66)
Anxiety (0–42)	8.8 ± 7.2	8.3 ± 5.1	0.0 (−6.0 to +6.0)	1.000	1.000	0.00 (−0.51, 0.49)
Stress (0–42)	13.6 ± 6.0	9.6 ± 5.2	+4.0 (−2.0 to +8.0)	0.067	0.200	0.45 (−0.06, 0.87)

Difference: Hodges–Lehmann location shift (post-traumatic minus congenital), the median of all pairwise differences. *q*: Benjamini–Hochberg adjusted value within each domain. δ: Cliff’s delta, with positive indicating higher scores in the post-traumatic group. All confidence intervals are 95% percentile bootstrap intervals from 5000 resamples stratified by aetiology. All intervals for the between-group difference include zero. No difference was detected in this sample; given its size, this is not evidence of equivalence.

**Table 3:** Correlates of child self-reported symptoms (RCADS-25 total).

	n	ρ (95% CI)	*p*	ρ|Age (95% CI)	*p*	*q*
**Family A—parental mental health**						
DASS-21 anxiety	24	0.79 (0.59, 0.90)	<0.001	0.78 (0.54, 0.89)	<0.001	<0.001
DASS-21 total	24	0.68 (0.37, 0.85)	<0.001	0.69 (0.34, 0.85)	<0.001	0.001
DASS-21 depression	24	0.50 (0.01, 0.86)	0.014	0.41 (−0.15, 0.85)	0.049	0.049
DASS-21 stress	24	0.38 (−0.06, 0.73)	0.067	0.43 (0.04, 0.73)	0.042	0.049
**Family B—orthopaedic function**						
MEPS	24	−0.47 (−0.74, −0.12)	0.021	−0.36 (−0.64, 0.02)	0.091	0.499
Surgical procedures, n	24	−0.31 (−0.64, 0.16)	0.147	−0.11 (−0.47, 0.33)	0.606	0.606
Total shoulder rotation	24	0.01 (−0.45, 0.45)	0.975	−0.14 (−0.51, 0.25)	0.535	0.606
Shoulder external rot.	24	−0.05 (−0.48, 0.40)	0.818	−0.22 (−0.59, 0.27)	0.317	0.499
Elbow flexion	24	0.19 (−0.23, 0.60)	0.373	0.21 (−0.24, 0.57)	0.333	0.499
Elbow extension deficit	24	−0.04 (−0.43, 0.39)	0.847	0.22 (−0.16, 0.54)	0.309	0.499
**Family C—clinical and other**						
CD-RISC-10	24	−0.84 (−0.93, −0.62)	<0.001	−0.74 (−0.90, −0.37)	<0.001	<0.001
KIDSCREEN-10	24	−0.60 (−0.90, −0.14)	0.002	−0.42 (−0.83, 0.11)	0.046	0.091
Age at diagnosis	24	−0.42 (−0.76, 0.04)	0.043	−0.30 (−0.65, 0.09)	0.168	0.168
Time since diagnosis	24	−0.10 (−0.48, 0.32)	0.642	0.35 (−0.10, 0.68)	0.099	0.132

Spearman’s ρ; ρ|age is the partial rank correlation adjusted for age (1 covariate, 21 degrees of freedom for the test). Confidence intervals are 95% percentile bootstrap intervals from 5000 resamples with the random seed fixed; within each resample for a partial correlation, the rank transformation and the residualisation were recomputed. Resamples in which a variable became constant, leaving the correlation undefined, were discarded; fewer than 15 of 5000 were affected for any variable. *q*: Benjamini–Hochberg adjustment applied to age-adjusted *p* values within each domain. The intervals are wide throughout, and interpretation rests on the estimates and their precision rather than on whether a threshold was crossed. All analyses are exploratory.

## Data Availability

The de-identified dataset and the analysis code are available from the corresponding author on reasonable request.

## Supplementary Materials

The following supporting information can be downloaded at: https://www.mdpi.com/article/10.3390/children13101371/s1.

## Abbreviations

CD-RISC-10	Connor–Davidson Resilience Scale, 10-item version
DASS-21	Depression Anxiety Stress Scales, 21-item version
FDR	False discovery rate
HRQoL	Health-related quality of life
ICC	Intraclass correlation coefficient
IQR	Interquartile range
MEPS	Mayo Elbow Performance Score
RCADS-25	Revised Children’s Anxiety and Depression Scale, 25-item version
RCADS-25-CG	Revised Children’s Anxiety and Depression Scale, 25-item caregiver version
SD	Standard deviation
