# Supplementary material for: Psychological Symptoms in Children with Radioulnar Synostosis: Associations with Self-Appraised Resilience, Parental Anxiety and Measured Elbow and Shoulder Function in a Cross-Sectional Multi-Informant Study
Source: Children (Basel). 2026 Oct 9;13(10):1371. doi: 10.3390/children13101371 (PMC13702892; doi:10.3390/children13101371)
Supplement: Supplementary file 1 [file children-13-01371-s001.zip › children-4543635-supplementary.pdf]

Supplementary Materials

The following are available: Table S1, sensitivity analysis of age associations using raw versus reference-standardised RCADS-25 scores; Table S2, child–parent concordance; Table S3, internal consistency of each instrument; Table S4, descriptive summary of the whole sample; Table S5, inter-rater reliability of the orthopaedic measures; Table S6, inventory of all analyses reported; Table S7, supporting detail for the exploratory subgroup analysis; Figure S1, total shoulder rotation by aetiology; Figure S2, total shoulder rotation and self-appraised resilience, Figure S3, self-appraised resilience and child-reported symptom scores.

Table S1. Age associations, raw versus age-normed scores.

Measure	Raw ρ	*p*	T-score ρ	*p*
Child RCADS-25 total	−0.61	0.002	−0.35	0.094
— Total Anxiety	−0.60	0.002	−0.24	0.259
— Total Depression	−0.54	0.006	−0.36	0.082
Parent RCADS-25-CG total	−0.44	0.032	−0.45	0.027
KIDSCREEN-10 (no norms applied)	0.52	0.010	—	—
CD-RISC-10 (no norms applied)	0.61	0.002	—	—

Table S2. Child–parent concordance.

Measure	Child	Parent	Difference	*p*	ρ	ρ | age
RCADS-25 total	20.0 ± 8.3	20.1 ± 7.7	+0.1	0.735	0.80	0.75
— Total Anxiety	12.2 ± 5.0	11.8 ± 4.7	−0.4	0.306	0.84	0.82
— Total Depression	7.8 ± 3.9	8.3 ± 3.7	+0.5	0.222	0.64	0.55
KIDSCREEN-10	35.1 ± 6.1	34.7 ± 6.7	−0.5	0.624	0.69	0.65

Difference is the mean parent minus child raw score; *p* is the Wilcoxon signed-rank test for the within-dyad difference; ρ is the Spearman correlation between informants.

Table S3. Internal consistency.

Instrument	Cronbach's α
RCADS-25 child, 25 items	0.859
— Total Anxiety, 15 items	0.747
— Total Depression, 10 items	0.770
KIDSCREEN-10 child	0.810
CD-RISC-10 child	0.896
RCADS-25-CG parent, 25 items	0.830
KIDSCREEN-10 proxy	0.861
DASS-21 total, 21 items	0.888
— Depression subscale, 7 items	0.828
— Anxiety subscale, 7 items	0.723
— Stress subscale, 7 items	0.705

Table S4. Descriptive summary, whole sample (n = 24).

Measure (range)	n	Mean	SD	Median	Range
RCADS-25 total (0–75)	24	20.0	8.3	20.0	6–32
— Total Anxiety (0–45)	24	12.2	5.0	13.0	2–19
— Total Depression (0–30)	24	7.8	3.9	7.0	0–14
KIDSCREEN-10 (10–50)	24	35.1	6.1	38.0	25–44
KIDSCREEN-10 child, T-score	24	43.1	6.5	45.7	32.9–55.1
CD-RISC-10 (0–40)	24	20.5	7.3	20.5	8–36
RCADS-25-CG total (0–75)	24	20.1	7.7	21.0	7–34
— CG anxiety (0–45)	24	11.8	4.7	13.0	4–19
— CG depression (0–30)	24	8.3	3.7	9.0	0–15
KIDSCREEN-10 proxy (10–50)	24	34.7	6.7	36.0	24–48
KIDSCREEN-10 proxy, T-score	24	41.2	10.9	41.7	26.1–70.4
Global health perception (1–5)	24	2.9	1.1	3.0	1–5
DASS-21 depression (0–42)	24	8.8	7.4	8.0	0–24
DASS-21 anxiety (0–42)	24	8.5	5.9	10.0	0–24
DASS-21 stress (0–42)	24	11.2	5.8	12.0	0–18

Table S5. Inter-rater reliability of the orthopaedic measures.

Measure	ICC(2,1)	ICC(2,2)
Elbow flexion	0.91	0.95
Elbow extension deficit	0.92	0.96
Shoulder external rotation	1.00	1.00
Total shoulder rotation	0.92	0.96
MEPS	0.76	0.86

Two-way random-effects intraclass correlation coefficients for absolute agreement, from the two examiners' independent measurements of each participant. ICC(2,1) describes the reliability of a single examiner's measurement; ICC(2,2) that of the mean of the two measurements, which was the value used in the analyses, and was obtained from ICC(2,1) by the Spearman–Brown formula. Shoulder flexion and abduction are omitted: both were at the same value in 22 of 24 participants, leaving too little between-participant variance for a reliability coefficient to be meaningful. MEPS, Mayo Elbow Performance Score.

Table S6. Inventory of all analyses reported.

Location	Analysis	Tests	Method	Correction
Table 1	Group comparisons, clinical and demographic	15	Mann–Whitney U, Fisher's exact	none
Table 2	Group comparisons, psychometric	13	Mann–Whitney U	BH within 3 domains

Location	Analysis	Tests	Method	Correction
Section 2.5, 3.2	Psychometric group comparisons repeated with age as a covariate	13	rank regression, group term tested	BH within 3 domains
Table 3	Correlates of child symptom scores, unadjusted and age-adjusted	14 × 2	Spearman, partial rank	BH within 3 domains
Section 3.2	KIDSCREEN-10 T-scores against the reference mean of 50	2	one-sample Wilcoxon signed-rank test (t test as a check)	none
Section 3.3	Resilience against parent-reported symptoms	2	Spearman, partial rank	none
Section 3.4	Robustness of the parental anxiety association	5 estimates	partial rank, leave-one-out	not applicable, same association
Section 3.5	Exploratory subgroup: shoulder rotation against five psychometric outcomes	5	Spearman	BH within subgroup family
Section 3.6	Time since diagnosis and symptoms, post-traumatic subgroup	2	Spearman, partial rank	none
Section 4.7	Parental education and responding parent's sex against the principal associations	6	Spearman, Mann–Whitney, partial rank	none
Table S1	Age associations, raw versus reference-standardised	10	Spearman	none
Table S2	Child–parent concordance	4 × 3	Wilcoxon, Spearman, partial rank	none
Table S3	Internal consistency	11 estimates	Cronbach's alpha	not applicable
Table S4	Descriptive summary	descriptive	—	not applicable
Table S5	Inter-rater reliability	5 × 2	ICC(2,1), ICC(2,2)	not applicable
Table S7	Exploratory subgroup, supporting detail	7 estimates	Spearman, partial rank, leave-one-out	see Section 3.5

The entries above comprise 108 hypothesis tests with p values and a further 33 estimates reported without hypothesis tests (5 in Section 3.4, 11 internal-consistency coefficients in Table S3, 10 reliability coefficients in Table S5 and 7 in Table S7), giving 141 tests and estimates in total. Table S4 is purely descriptive and is not counted. All are exploratory. No analysis plan was registered in advance, and the groupings used for multiplicity correction

were made when the analyses were assembled rather than before data collection. BH, Benjamini–Hochberg false discovery rate procedure.

Table S7. Shoulder rotation and self-appraised resilience within the congenital subgroup (n = 14).

Analysis	ρ	*p*
Unadjusted	0.62	0.018
Adjusted for age	0.62	0.024
Adjusted for age and MEPS	0.60	0.039
Post-traumatic subgroup (n = 10), unadjusted	−0.21	0.556
Whole sample (n = 24), unadjusted	0.10	0.628
Leave-one-out range, unadjusted	0.54 to 0.73	—
Leave-one-out range, age-adjusted	0.51 to 0.73	—

Within the congenital subgroup neither shoulder rotation (ρ = 0.05) nor CD-RISC-10 (ρ = 0.13) was correlated with age. Shoulder rotation and MEPS were correlated within this subgroup (ρ = 0.65, $p = 0.012$), whereas MEPS and CD-RISC-10 were not (ρ = 0.26, $p = 0.366$). Across the five psychometric outcomes examined against shoulder rotation in this subgroup the association did not survive correction for multiplicity ($q = 0.091$). This analysis is exploratory and hypothesis-generating; with fourteen observations and five tests it should not be interpreted as a finding.

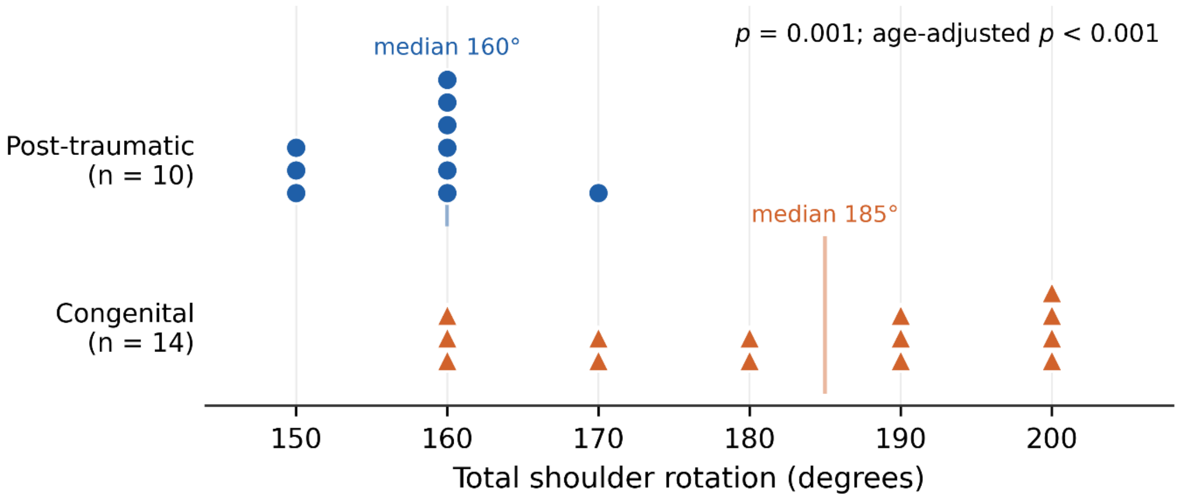

Figure S1. *Total shoulder rotation by aetiology (n = 24).* Individual values in degrees, stacked where they coincide; vertical lines mark group medians. Congenital cases showed greater total shoulder rotation with little overlap between distributions. Unlike every psychometric comparison, the difference was independent of age (age-adjusted $p < 0.001$; correlation with age ρ = −0.19, $p = 0.377$).

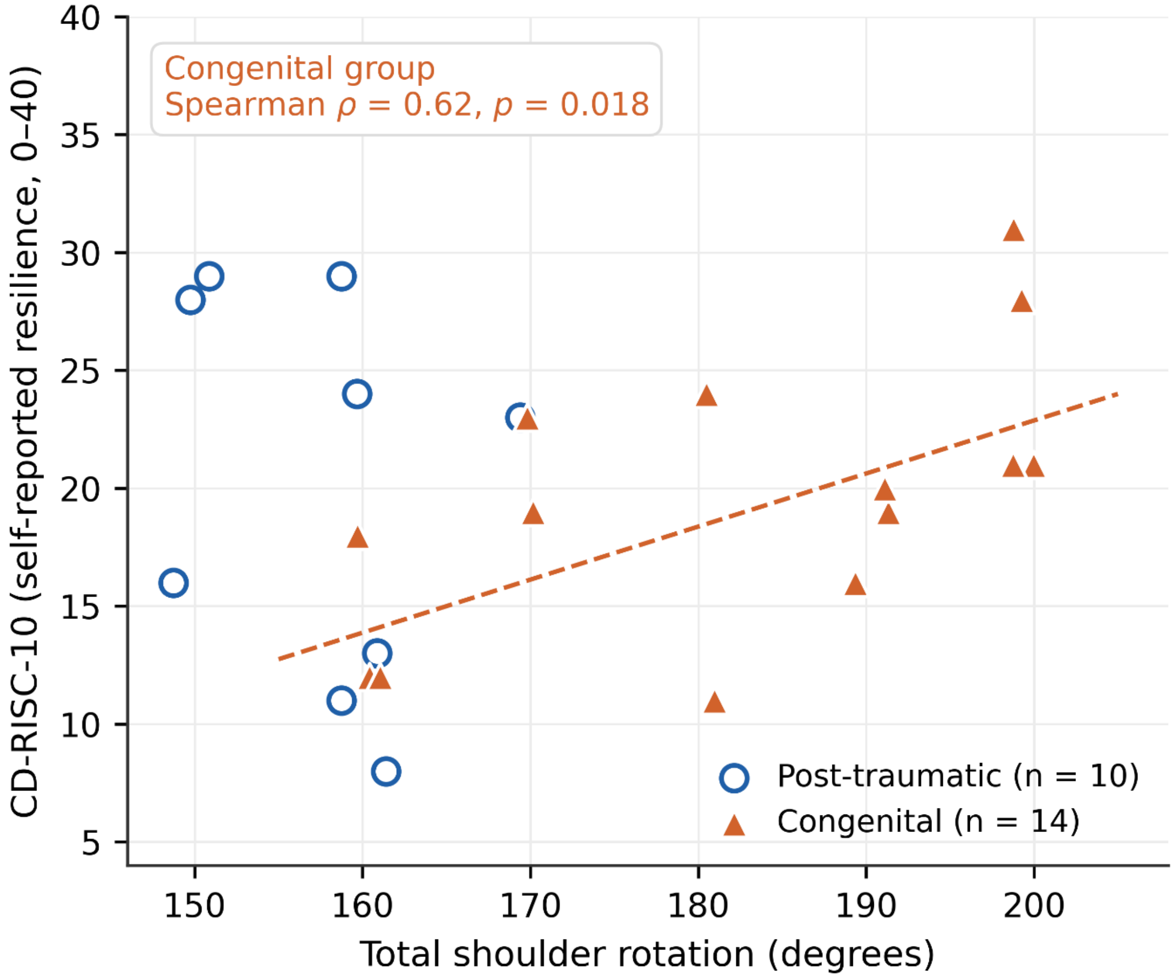

Figure S2. *Total shoulder rotation and self-appraised resilience (n = 24).* Filled triangles denote congenital cases (n = 14), open circles post-traumatic cases (n = 10). The dashed line is a Theil–Sen robust fit for the congenital group, shown for orientation; the reported statistic is Spearman's ρ. A small horizontal jitter (± 1.4°) separates coincident points. The association was independent of age within the congenital group (age-adjusted partial ρ = 0.62, $p = 0.024$) while the corresponding estimate in the post-traumatic subgroup was ρ = −0.21. It did not survive correction across the five psychometric outcomes examined ($q = 0.091$) and is reported as exploratory.

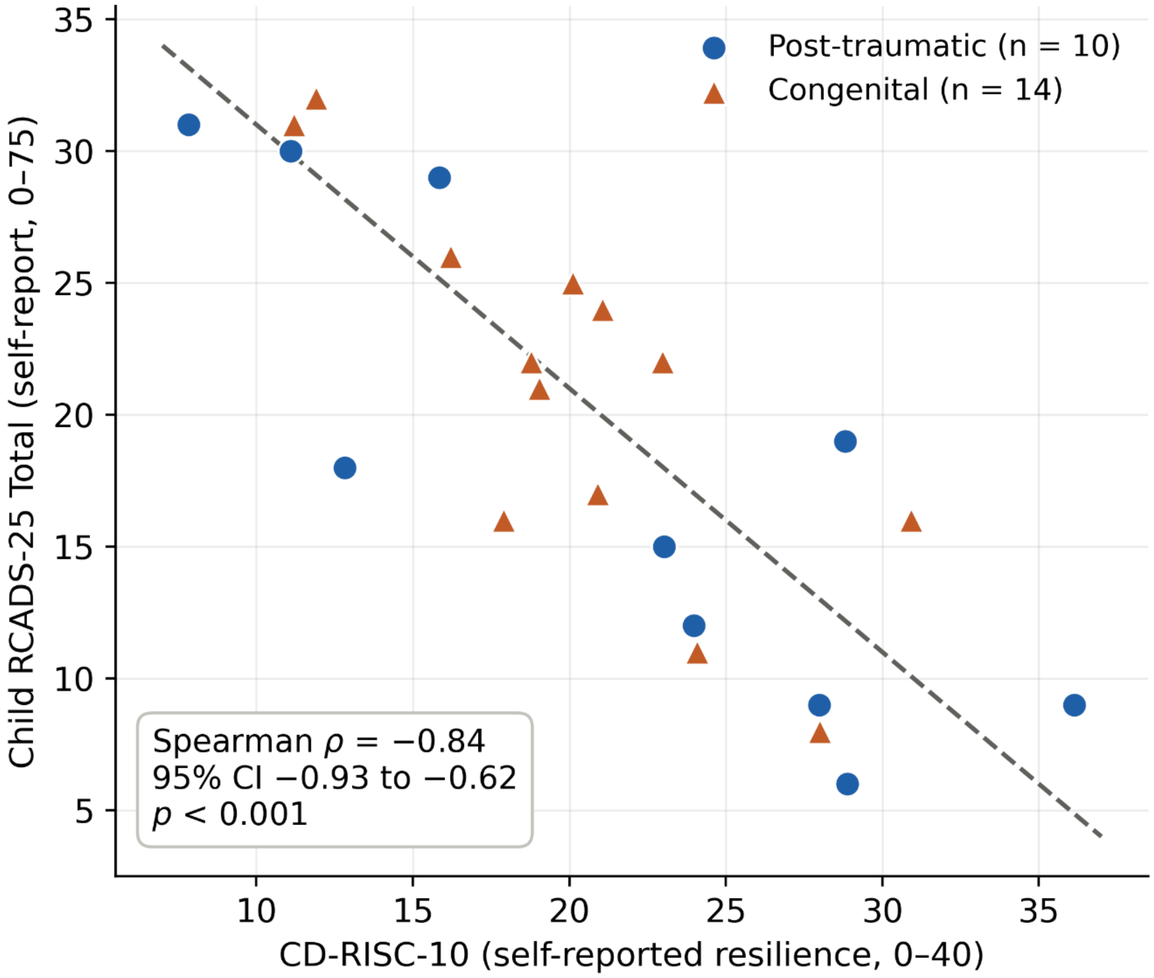

Figure S3. *Self-appraised resilience and child-reported symptom scores (n = 24).* Circles denote post-traumatic cases (n = 10), triangles congenital cases (n = 14). The dashed line is a Theil–Sen robust fit shown for orientation; the reported statistic is Spearman's ρ. A small horizontal jitter (± 0.22) separates coincident points. Both measures were completed by the child, so same-informant method variance cannot be excluded (Section 4.3).
